# Shifts in the Skin-Associated Microbiota of Hatchery-Reared Common Snook *Centropomus undecimalis* During Acclimation to the Wild

**DOI:** 10.1007/s00248-018-1252-7

**Published:** 2018-09-06

**Authors:** Andrea M. Tarnecki, Nathan P. Brennan, Ryan W. Schloesser, Nicole R. Rhody

**Affiliations:** 1 Mote Marine Laboratory, 1600 Ken Thompson Parkway, Sarasota, FL 34236 USA; 2 Mote Aquaculture Research Park, 874 WR Mote Way, Sarasota, FL 34240 USA

**Keywords:** Common snook, *Centropomus undecimalis*, Microbiota, Stock enhancement, Immunity

## Abstract

The skin-associated microbiota of fish competes against pathogens for space and nutrients, preventing colonization by harmful bacteria encountered during environmental transitions such as those faced during stock enhancement. Thus, alterations in bacterial community structure during release of cultured fish have important implications for health of these individuals. This study investigated microbiota structure during acclimation of juvenile hatchery-reared common snook *Centropomus undecimalis* to the wild by comparing skin-associated microflora among snook in captivity, after 48 h of acclimation at release sites, and from the wild. After two days of acclimation, the microbiota of hatchery-reared snook mirrored that observed on wild snook. Relative abundances of potential pathogens were higher in captive fish, whereas acclimated and wild fish harbored bacterial taxa influenced by geographical factors and water quality at release sites. Predicted microbiota function of acclimated and wild fish showed higher production of protective amino acids and antimicrobials, identifying a mechanism for microbial supplementation of the immune defense of these fish. The two-day transition to wild-type microbiota suggests a temporal scale of hours associated with bacterial succession indicating that the microbiota, whose structure is vital to fish health, aids in acclimation of fish to new environments during stock enhancement efforts.

**Electronic supplementary material:**

The online version of this article (10.1007/s00248-018-1252-7) contains supplementary material, which is available to authorized users.

## Introduction

Common snook *Centropomus undecimalis*, a catadromous estuarine species of the Western Atlantic, is a marine sportfish that contributes to the Florida marine recreational fishery whose sales alone are valued at $10 billion each year [1]. However, sensitivity to cold and red tide as well as habitat loss have historically threatened the fishery; thus, the species is a prime candidate for stock enhancement efforts. There is an active stocking program for common snook to test the effectiveness of stock enhancement as a fishery management tool and develop techniques to optimize post-release survival. Brennan et al. [2] demonstrated a 1.78× increase in survival by allowing juvenile snook to acclimate within predator-free enclosures for three days prior to release into estuarine environments. The authors hypothesized that increased survival was primarily due to learning predator avoidance, but the fish also benefit from learning to feed in the wild and recovering from the handling and stress associated with transport from the hatchery to the release sites.

In addition to behavioral and physiological adaptations during and post-release, the innate immune system of fish must be capable of quickly responding to the bacterial species unique to the new environment. Microbial communities, termed microbiota, associated with fish play a significant role in the health of their host, including increasing nutrient availability, boosting immune function, and protecting against opportunistic pathogens through competitive exclusion and production of antimicrobials [3, 4]. Microbiota structure is intimately tied to health and immune function, as stress and disease correlate with a decrease in bacterial diversity and a concurrent increase in the proportion of opportunistic pathogens [5, 6], and exposure to beneficial microorganisms can result in increased mucosal (skin, gill, gastrointestinal) immunity [7]. Immunity at mucosal surfaces is of great importance in fish as it provides the first barrier between the host and environmental pathogenic microorganisms.

Rearing fish in captivity or bringing them into a culture environment from the wild drastically alters the fish microbiota. These differences include decreases in bacterial diversity and changes in taxonomical structure [8–11]. Larval colonization by commensal bacteria guides the development of the fish innate immune system [12]; thus, colonization of fishes by a less diverse bacterial assemblage during rearing in culture systems may offer more available niche environments for pathogen establishment, as well as negatively impact the ability of the immune system to resist infection and disease. Characterizing the changes in microbiota structure during acclimation is a vital first step towards understanding disease susceptibility in newly released individuals. Studies comparing microbiota composition and immune status between cultured, acclimated, and wild fish are absent from the literature, and to our knowledge, the ability of the microbiota of captive fish to adapt to the wild environment is unknown.

This study aimed to develop and employ a replicable approach to investigate shifts in microbiota structure and innate immune parameters during wild acclimation of juvenile hatchery-reared common snook in order to increase understanding of the benefit of an acclimation period to post-release survival.

## Methods

### Snook Rearing and Release

Snook reared in this study came from two separate spawning events. The first spawn originated from captive adult broodstock held and maintained at the Mote Aquaculture Research Park (MAP). Broodfish at MAP were maintained in large (48,000 L) indoor tanks and induced to spawn as previously described [13, 14]. The second spawn originated from strip spawning of wild adult common snook from Tampa Bay (Florida, USA) as previously described [15]. Regardless of spawn source (wild vs captive spawned fish), eggs hatched at MAP in three separate 100 L conical tanks. Newly hatched larvae were subsequently transferred from the egg hatchers and volumetrically stocked (200 L^−1^) into individual rearing tanks (3.3 m^3^). The fish were cultured using standard protocols [16] and remained in a recirculating aquaculture system until the time of tagging and release. Fish from the first spawn were released at 186 days post-hatch (dph), and fish from the second spawn were released at 117 dph. One month prior to release, all juvenile snook (85–158 mm total length, TL) were fitted with coded wire tags to provide a unique identification of the cohort and release location for each fish as in Brennan et al. [17]. After tagging, snook were graded and allocated in rearing tanks by size, smalls (56–116 mm TL) and larges (117–158 mm TL). At the time of release, a live hauler transported fish to two tidal creeks located in Sarasota, Florida, USA: Whitaker bayou (27.357 N, 82.547 W) and Hudson bayou (27.327 N, 82.536 W). A total of 50 randomly selected snook were stocked into a single cage enclosure (1 m^3^) in each creek. This enclosure allowed them to acclimate to the natural environment for 48 h [2] prior to sample collection and release into the creeks.

### Sample Collection

Three groups of juvenile snook were sampled during the course of the study. Hatchery-reared fish from two separate spawning events were sampled in the hatchery on the day prior to release and will be referred to herein as captive fish (Capt1 and Capt2). As the sampling procedure has the potential to impact the microbiota structure of these individuals, these fish were then moved to a separate holding tank so as not to include them in acclimation cages and future sampling. Following acclimation, individuals were removed and sampled from cages for each spawn at each location immediately prior to release (acclimated fish from hatch1: Whitaker [Accl1-W] and Hudson [Accl1-H]; acclimated fish from hatch2: Whitaker [Accl2-W] and Hudson [Accl2-H]). During and between release events, seine nets were used to catch and sample wild juvenile snook of a similar size as those intended for release from the same locations (Wild-Whitaker [Wild-W], Wild-Hudson [Wild-H]). Hatchery-reared fish from the first spawn were placed into acclimation cages on August 3, 2016, and fish from the second spawn were placed on October 5, 2016. Wild fish were sampled from July 28 to August 23, 2016. A YSI ProPlus multimeter (YSI, Yellow Springs, OH, USA) recorded water quality at each sampling location (initial hatchery tanks, acclimation sites, and wild fish capture sites). Water parameters measured included the following: salinity (psu), temperature (°C), dissolved oxygen (mg L^−1^), pH, and turbidity (FNU).

In order to obtain sufficient volumes of external mucus to perform the desired tests, the mucus from three individual snook was pooled to make one sample. A sterile spatula removed external mucus by gentle scraping, and mucus was held on ice until arrival at the laboratory (~ 4 h). Each fish was measured (TL, mm) following mucus sampling. Samples were gently mixed, and a sterile cotton swab was used to sample a portion of the mucus for microbiota analysis. Remaining mucus was centrifuged at 2,630 x g at 4 °C for 15 min to remove debris [18] and divided into smaller aliquots for immune parameter measurements. The aliquots and swabs were stored at − 80 °C until further analysis.

### Sample Processing

DNA was extracted from swabs using the PowerSoil® DNA Isolation Kit (MO BIO Laboratories, Inc., Carlsbad, CA, USA) following manufacturer’s instructions. PCR was performed on the V4 variable region of the 16S rRNA gene using the HotStarTaq Plus Master Mix Kit (Qiagen, Valencia, CA, USA) and the primers 515F/806R under the following conditions: 94 °C for 3 min, followed by 28 cycles at 94 °C for 30 s, 53 °C for 40 s, and 72 °C for 1 min, with a final extension step of 72 °C for 5 min. PCR products were pooled in equal proportions based on molecular weight and DNA concentration, purified with calibrated Ampure XP beads, and sequenced using the Illumina MiSeq platform (Illumina, Inc., San Diego, CA, USA) following standard protocols. Sequencing was performed at MR DNA (www.mrdnalab.com, Shallowater, TX, USA). Resulting sequences were processed using the MiSeq SOP ([19], accessed 1 Mar 2017) in Mothur v.1.38.1 ([20], accessed 8 December 2017). Operational taxonomic units (OTUs) were defined at 97% sequence similarity [21] and classified to the lowest taxonomic level using SILVA [22] and a bootstrap cut-off of 50%. Mothur was used to calculate Good’s coverage, diversity indices (bacterial richness as defined by total number of OTUs, and Shannon’s Evenness Index (SEI)), and rarefaction curves. The resulting OTU abundance table was loaded into Primer v6 [23] for nonparametric statistics to allow for comparisons between fish groups.

Innate immune parameters analyzed in this study included superoxide dismutase (SOD) and alkaline phosphatase (AKP). Mucus samples were assayed for total protein using the Bradford Protein Assay (Bio-Rad Laboratories, Inc., Hercules, CA, USA) based on the method by Bradford [24]. SOD activity was measured using the Superoxide Dismutase Assay Kit (Cayman Chemical, Ann Arbor, MI, USA) which quantifies the activity of the three types of SOD (cytosolic Cu/Zn, mitochondrial Mn, and extracellular FeSOD). AKP activity was measured as described by Subramanian et al. [25]. Enzyme activities were adjusted for total protein content.

### Data Analysis

Number of sequences per sample were rarefied to the sample with the least number of sequences (14,641) prior to calculation of diversity indices and rarefaction curves. Diversity indices (richness, evenness), enzyme activities (SOD, AKP), and TL were compared among fish groups (captive, acclimated, wild) using analysis of variance (ANOVA). Microbiota composition was compared among captive, acclimated, and wild juvenile snook using permutational analysis of variance (PERMANOVA) and analysis of similarities (ANOSIM), and visualized using multidimensional scaling (MDS). OTUs responsible for differences between fish groups were determined using LDA effect size (LEfSe) [26]. Correlations between bacterial taxa and innate immune activity were determined using partial least square (PLS) regression and visualized using clustered image maps (CIM) in the mixOmics package [27] within R [28]. Predictive functions of the microbial communities were determined using predictive functional profiling of microbial communities (PICRUSt) [29]. Briefly, OTUs were classified using Greengenes [30] database files from May 2013 in Mothur, and a biom file was generated. Data was uploaded into Galaxy (huttenhower.sph.harvard.edu/galaxy/, accessed 9 May 2017) where it was normalized by copy number, and the functional metagenome was predicted using KEGG Orthologs and categorized by function at KEGG Pathway Hierarchy Level 3. Accuracy of PICRUSt predictions was evaluated using the nearest sequence taxon index (NSTI) [29]. The resulting file was loaded into statistical analysis of metagenomic profiles (STAMP) software v2.1.3. Non-bacterial functions were removed (i.e., Human Diseases and Organismal Systems). Differences among groups (captive, acclimated, wild) were determined by ANOVA followed by Tukey–Kramer post-hoc tests. KEGG pathways were filtered by *p* value (*p* < 0.05) and effect size (eta^2^ > 0.70).

#### Data Availability

The datasets generated during and analyzed during the current study are available in the Sequence Read Archive repository, www.ncbi.nlm.nih.gov/sra/, SRA Study accession: SRP143622.

## Results

### Fish Size and Water Quality

No mortality occurred in the cages within the 48 h of acclimation. Total lengths of juvenile snook ranged from 62 to 266 mm with an overall average of 158 mm (Table 1). Fish from spawn 2 and wild fish from Whitaker bayou were significantly shorter than fish from spawn 1. Salinity and dissolved oxygen were slightly higher in initial hatchery tanks than corresponding release and wild sampling sites (Table 2). Dissolved oxygen was generally lower at acclimation sites than in hatchery tanks and wild sites. Turbidity varied between sampling sites.

**Table 1:** Total length ± standard deviation (mm) of fish in this study. Superscripts denote significance as determined using ANOVA (*α* = 0.05)

Fish group	Total individuals sampled (number of replicates)	Total length (mm ± SD)
Capt1	30 (10)	198 ± 18.2^a^
Capt2	24 (8)	124 ± 19.7^c^
Accl1-H	30 (10)	195 ± 17.3^a^
Accl2-H	24 (8)	141 ± 6.26^bc^
Wild-H	24 (8)	164 ± 33.6^ab^
Accl1-W	30 (10)	200 ± 17.5^a^
Accl2-W	24 (8)	121 ± 12.7^c^
Wild-W	24 (8)	123 ± 55.4^bc^

**Table 2:** Water quality parameters measured at each sampling point

Sample	Salinity (psu)	Temperature (°C)	Dissolved oxygen (mg L^−1^)	pH	Turbidity (FNU)
Capt1	32.30	28.85	6.63	8.17	–
Capt2	34.55	27.30	6.44	8.59	–
Accl1-H	32.58	31.30	3.00	7.94	15.80
Accl2-H	30.34	28.30	2.93	8.05	180.6
Wild-H	30.60	32.05	6.01	8.05	20.55
Accl1-W	28.18	30.95	2.91	7.78	4.700
Accl2-W	27.88	29.30	2.78	8.06	183.0
Wild-W	28.39	33.68	7.26	7.99	30.79

### Microbiota Diversity

**Fig. 1:**
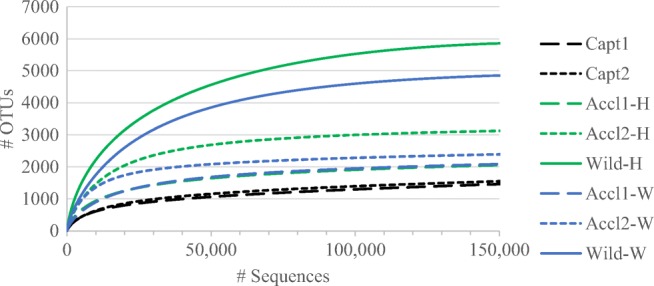
Rarefaction curves from sequencing of the skin microbiota of common snook juveniles

Sequencing resulted in 2,649,228 assembled contigs. Further sequence processing included the following: optimization of sequence length (removal of sequences that ended before the position that 90% of the sequences ended), removal of sequences with ambiguous base calls, removal of chimeras and singletons, and removal of sequences classified into nonbacterial lineages. Remaining sequences (1,663,645 sequences remained following processing) contained 12,806 OTUs at 97% sequence similarity, ranging from 180 to 2214 OTUs per sample, with an average of 647. Sequence coverage was > 96% for all fish groups as indicated by Good's coverage and rarefaction curves (Fig. 1), suggesting that our sequencing efforts detected the majority of fish-associated bacterial community members.

**Fig. 2:**
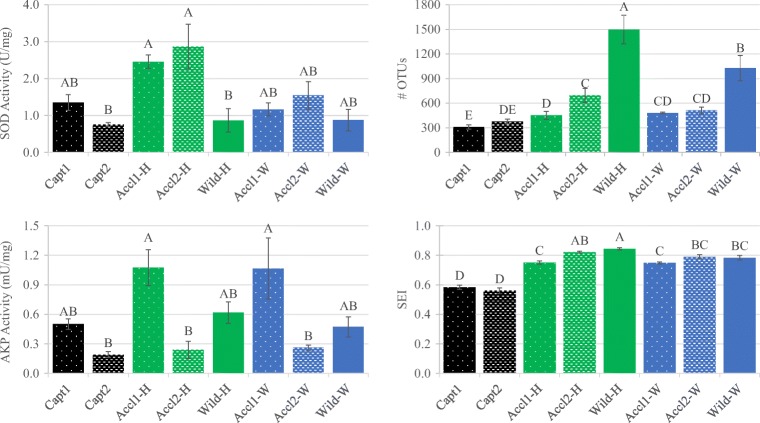
Innate immune parameters and diversity indices of common snook juveniles. Richness was determined by number of OTUs and evenness by Shannon’s Evenness Index. Significance as determined by ANOVA is indicated by letters

Bacterial richness was significantly higher in wild samples than in captive and acclimated fish (Fig. 2). Wild fish from Hudson bayou harbored higher richness than wild fish from Whitaker bayou. Acclimated fish generally harbored similar diversity to captive fish, with some significant increases, suggesting a trend towards increased diversity during the acclimation period. These results were similar for evenness (SEI), with captive samples having less even bacterial distributions than wild fish. However, evenness indices were all significantly higher in acclimated fish as compared to initial captive fish, indicating better integration with wild fish in this measurement as opposed to species diversity.

### Microbiota Structure

**Fig. 3:**
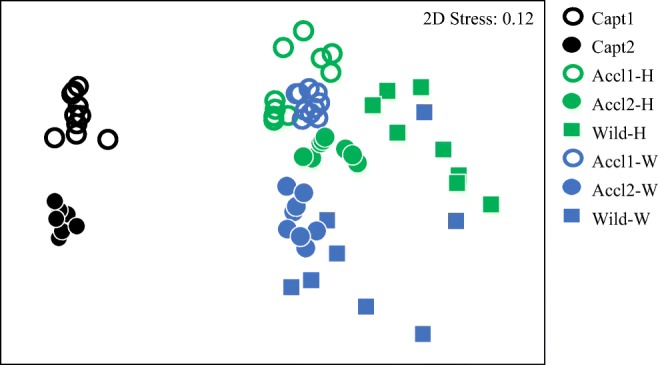
Multidimensional scaling plot indicating similarity between skin microbiota structures of common snook juveniles

Microbiota structure was significantly different between each of the eight sample groups as determined by PERMANOVA (pairwise *p* ≤ 0.002) and ANOSIM (global *p* = 0.001, global *R* = 0.888) (Fig. 3). Pairwise tests within ANOSIM indicated variation in community overlap depending on sample group. For example, Wild-W and Accl2-W had the lowest *R* value at 0.411, suggesting the greatest similarity in microbial community structure between acclimated and wild fishes, whereas greater separation occurred between captive fish and post-release groups (*R* values > 0.995).

Proteobacteria dominated the microbiota of all fish groups. However, microbiota structure varied at the phylum level with initial captive samples having higher abundances of Firmicutes and lower abundances of Bacteroidetes and Actinobacteria. Acclimation generally resulted in increases in the groups more largely represented in wild fishes paired with decreases in Firmicutes. Wild fish collected from both Whitaker bayou and Hudson bayou showed similar overall phylum-level patterns. Captive fish from the second spawn had higher abundances of Firmicutes coupled with lower abundances of Proteobacteria than captive fish from the first spawn. Acclimated fish from spawn 1 had less representatives from Chloroflexi and Planctomycetes as compared to their captive and wild counterparts.

**Fig. 4:**
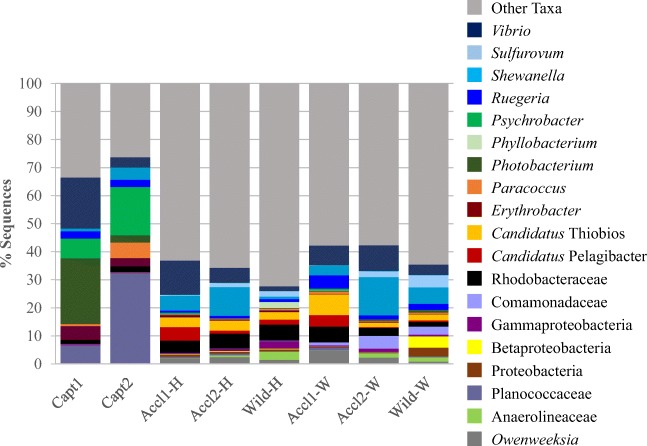
Relative abundance by percent of lowest possible taxonomic classification identified in sequencing of the skin microbiota of juvenile common snook

At the lowest taxonomic classification, differences between fish groups were apparent (Fig. 4) and LEfSe identified 58 differential OTUs within 46 taxa between groups (Table 3). Core members of the microbiota (present in all 70 samples) included OTUs classified within many of the most abundant taxa including *Vibrio* (1 OTU), *Photobacterium* (1 OTU), *Psychrobacter* (1 OTU), *Candidatus* Thiobios (1 OTU), *Ruegeria* (1 OTU), *Tropicimonas* (1 OTU), Planococcaceae (1 OTU), Rhodobacteraceae (2 OTUs), Comamonadaceae (1 OTU), Oceanospirillales (1 OTU), and Proteobacteria (1 OTU). These core OTUs made up 5.9–70.5% of total sequences per sample, with captive fish typically having more (averaging about 55% total sequences) sequences falling into the core microbiota than wild (averaging 14% total sequences) and acclimated (averaging 21% total sequences) fish. On the other hand, all eight fish groups shared 273 OTUs, and sequences attributed to these OTUs made up 72.5% of total sequences identified in this study. Of these, the most abundant (> 5% total sequences standardized to fish group with least number of sequences) include *Vibrio*, *Shewanella*, and Planococcaceae. Interesting to note is that only 32.6% of all sequences could be classified confidently to the genus level using the processing method in this study, including the relatively liberal bootstrap value of 50%.

**Table 3:** Number of OTUs in each discriminatory taxon between fish groups as determined by LEfSe

OTU ID	Captive	Acclimated	Wild
*Acholeplasma*	0	0	1
*Acinetobacter*	0	1	0
*Acinetobacter lwoffii*	0	1	0
*Acinetobacter venetianus*	0	1	0
Alphaproteobacteria	0	1	0
*Arcobacter*	0	1	0
*Bacillus*	1	0	0
Bacteroidetes	0	0	1
*Balneola*	0	1	0
C111	1	0	0
Candidatus *Aquiluna rubra*	1	0	0
*Chryseomicrobium imtechense*	1	0	0
Comamonadaceae	0	1	0
Cryomorphaceae	0	3	1
*Desulfococcus*	0	1	1
*Desulfosarcina*	0	1	0
*Erythrobacter citreus*	1	0	0
Flavobacteriaceae	1	2	0
GMD14H09	0	1	0
Helicobacteraceae	0	1	1
*Kytococcus*	0	1	0
*Marinicella*	1	0	0
*Marinococcus*	1	0	0
*Massilia niastensis*	0	1	0
Microbacteriaceae	0	1	0
*Microbispora rosea*	0	1	0
*Oleibacter*	1	0	0
Oleiphilaceae	0	1	0
OM60	0	0	1
*Paracoccus zeaxanthinifaciens*	0	1	0
Pelagibacteraceae	0	2	0
*Persicirhabdus*	0	0	1
*Photobacterium damselae*	1	0	0
Planococcaceae	1	0	0
*Psychrobacter celer*	1	0	0
*Psychrobacter pacificensis*	1	0	0
Rhodobacteraceae	1	1	0
Rhodospirillaceae	0	2	0
Rhodospirillales	0	2	0
Saprospiraceae	0	2	0
SHA-20	0	0	1
*Spongiibacter tropicus*	0	1	0
*Sulfurovum lithotrophicum*	0	0	1
*Thalassomonas*	0	0	1
*Vesicomyosocius okutanii*	0	1	0
*Vibrio*	1	0	0

### Predicted Microbiota Function

**Fig. 5:**
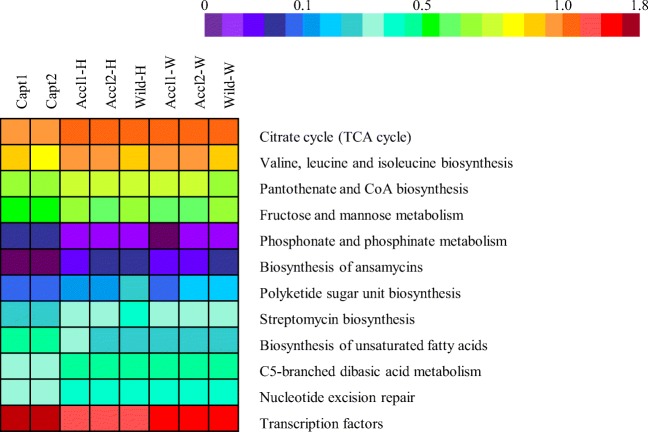
Heatmap of predicted metagenome functions of the skin microbiota of common snook juveniles as determined by PICRUSt analysis

NSTI scores averaged 0.11 ± 0.03, indicating the ability to use this data to interpret predicated microbial community function (an NSTI score of 0.17 was found to provide accurate metagenome predictions in soil samples) [29]. Twelve KEGG level 3 pathways differed significantly between groups with an effect size of > 0.70 (Fig. 5). The most significant differences included biosynthesis of unsaturated fatty acids (eta^2^ = 0.852) which was highest in captive fish, followed by acclimated and then wild fish, and streptomycin biosynthesis (eta^2^ = 0.828) which showed the exact opposite pattern. Other significant pathways higher in wild and acclimated fish included the following: metabolism of fructose and mannose and C5-branched dibasic acid, biosynthesis of polyketide sugar unit, pantothenate and CoA, valine, leucine and isoleucine, and ansamycins, nucleotide excision repair, and the citrate cycle. Pathways higher in captive fish included the following: phosphonate and phosphinate metabolism and transcription factors.

### Innate Immunity

Captive fish (Capt1, Capt2) demonstrated similar SOD activity as wild fish. SOD activity was significantly higher in acclimated snook in Hudson bayou than wild fish in Hudson bayou (Fig. 2). Although there was a trend for the same pattern in Whitaker, this trend was non-significant. AKP activity was highest in acclimated fish from spawn 1 irrespective of bayou; however, activity was generally lowest in acclimated fish from spawn 2. AKP activity correlated positively with TL (Spearman’s rank order correlation, coefficient = 0.553, *p* < 0.001), whereas SOD activity correlated less but still significantly with TL (coefficient = 0.283, *p* = 0.0179). PLS indicated that the highest correlations between bacterial taxa and fish-associated parameters (TL, AKP, SOD) were positive (> 0.6) between SOD and Armatimonadales, *Asticcacaulis* and *Bradyrhizobium* (Alphaproteobacteria), and *Xenophilus* (Betaproteobacteria). Four taxa identified as sulfur-metabolizing bacteria correlated negatively with TL, and the greatest negative correlation occurred between *Sulfurovum*, a sulfur-oxidizing bacterium, and TL. Only ML602J-51 (Actinobacteria) and *Candidatus* Pelagibacter (Alphaproteobacteria) correlated positively with TL at the coefficient examined.

## Discussion

### First Description of the Juvenile Snook Microbiota

This study provides the first description of the juvenile common snook skin microbiota, including the first characterization of the wild snook microbiome. Bacterial species richness detected in snook was generally higher than that of other fish species [31–33]. Fish exhibit high inter-individual variation in their microbiomes [34–37]; thus, pooling individuals likely contributed to higher richness. Despite pooling, inter-sample variation was high, particularly among wild fishes. Our sampling areas were tidal creeks inundated by both fresh and marine waters, with bacterial representatives from both ecosystems interacting with the snook mucosa. These converging environments likely contribute to the high diversity detected in this species.

Geographical location influenced the wild snook microbiota, as skin-associated bacteria differed between fish from Whitaker and Hudson bayous, and acclimated fish acquired bacteria similar to wild fish from their respective release site. However, wild snook shared 85% of sequences (2253 OTUs) despite sampling location. Interestingly, acclimated fish from spawn 2 demonstrated these geographical differences more so than those from spawn 1. These results support the hypothesis that microbiome assembly is complex and involves habitat filtering from external environmental parameters and the host (i.e., species-specific differences in mucus composition and immune function) as well as competition with previously established microorganisms [38]. Despite external influences on its structure [31, 39], the fish mucosal microbiome is highly divergent from that of the surrounding water [31, 38, 40]. Species-specificity of fish-associated bacterial communities [3, 34, 39] and influences of genetics on microbiota structure [33, 40] demonstrate the importance of host-related factors in determination of fish microbiota assembly.

Juvenile snook shared 12 OTUs across all samples (11 Proteobacteria, 1 Firmicutes). Proteobacteria dominates the core microbiota of fishes [9, 10, 31, 38, 40] suggesting an important relationship between this taxon and its hosts. Juvenile common snook share members of their core microbiota, including *Vibrio* and *Ruegeria*, with larval common snook [41], but other members were unique to juveniles, illustrating differences between snook microbiota among developmental stages. Members of the genera *Vibrio*, *Photobacterium*, and *Psychrobacter* are normal inhabitants of marine fish skin [31, 42, 43]. However, core members *Candidatus* Thiobios and *Tropicimonas* are unique to snook in this study, providing further evidence towards species-specificity of fish microbiomes. 16S rRNA sequences could not identify many shared OTUs to the genus or even family levels; thus, the skin-associated microbiota of common snook represents an underexplored environment for bacterial diversity.

Data from this study indicates that the snook microbiota transitions from a captive structure to wild-type at a temporal scale of hours. As such, sampling these fish after 2 days of acclimation likely missed several transitions in the external bacterial community structure, and better understanding of bacterial succession rates requires hourly assessments of the microbiota.

### Captivity Significantly Alters Microbiota Structure

Captive common snook juveniles harbored decreased bacterial species richness and evenness as compared to acclimated and wild snook. Numerous studies on the gut microbiota of fishes report similar findings [8, 9, 44, 45]. Captive fish rearing in a recirculating aquaculture system maintains a relatively constant salinity, temperature, and oxygen level, generally buffered by the external environment. However, both acclimated and wild fish encountered daily fluxes in salinity, consistently high temperatures, low oxygen levels, and increased turbidity from tidal creeks. The static nature of the culture environment likely plays a large role in the decreased diversity of bacteria associated with the external mucus layer. Conversely, freshwater and marine environments influence the microbiota of fish from tidal creeks, providing bacterial species from both environments with the opportunity to interact and establish on fishes.

Wild and acclimated fish encounter greater diet variability, feeding on living organisms as opposed to the pelleted feed given in captivity, supplying a wider assortment of nutrients that enrich for bacterial communities with diverse metabolic capabilities. The diet of fish also alters the composition of the skin microflora [46]. Indeed, enriched KEGG pathways identified in the microbiota of captive fishes (phosphonate/phosphinate metabolism and biosynthesis of unsaturated fatty acids) are concomitant with increased consumption of amino acids and glucose [47, 48] and indicate a potential interaction between alterations in diet and the structure of the snook skin microbiota. The influence of dietary modifications on the skin-associated bacteria deserves further attention, as Landeira-Dabarca et al. [46] documented a change in 7 days, but it is unknown if these influences are observable within 2 days.

Captive fish harbored greater abundances of bacterial groups known to contain opportunistic fish pathogens, particularly the genera *Vibrio* and *Photobacterium*. *Vibrio* contains 13 species that cause disease in marine fish and diseases resulting from *Photobacterium* impact multiple species of finfish and sharks [49], and these pathogens are of grave concern in aquaculture. However, 16S rRNA sequencing cannot always classify these organisms to the species level [50], and both genera are often reported in association with the external surfaces of fish [31, 42, 43]. The microbiota of captive fishes, including snook in this study and fine flounder *Paralichthys adspersus* [51], demonstrate reduced valine, leucine, and isoleucine biosynthesis. These amino acids aid in lymphocyte proliferation, and deficiencies increase susceptibility to pathogens [52]. Higher relative abundances of opportunistic taxa and decreased amino acid biosynthesis, alongside a decrease in overall bacterial diversity, warrant further investigation, including identification to the species level and studies that go beyond predictive function, to determine the implications for disease susceptibility in captive fish.

Despite concerns of an altered microbiota (dysbiosis) due to captive rearing, the ability of the bacterial communities to resemble wild-type assemblages within 2 days of acclimation suggests that dysbiosis can be overcome quickly. The newly adapted, diverse microbial community may colonize niches within the mucosal layer to aid in protection against pathogens encountered in the wild as the snook transition between environments.

### Innate Immune Activity and the Skin Microbiota Are Impacted by Acclimation

SOD activity was highest in juvenile snook during acclimation. SOD activity increases in fish exposed to chemical contaminants [53, 54], osmotic shock [55], and hypoxia [56]. The locations in this study were similar in salinity and temperature to those found in the hatchery, but acclimation sites measured the lowest dissolved oxygen levels and the highest SOD activity. Thus, environmental conditions, including oxygen saturation or other unmeasured parameters, influence SOD activity in these fish. On the other hand, patterns in AKP activity in this study correlated well with fish length, indicating that common snook express AKP constitutively and activity increases with fish size.

Bacterial taxa associated with acclimating and/or wild snook express KEGG pathways significant to immunity. Biosynthesis of valine, leucine, and isoleucine, whose protective role against infections was discussed earlier, was highest in acclimated fish, zebrafish experiencing osmotic stress [57] and oil-exposed flounder [58], indicating that environment and contaminants alter the microbial community in a way that increases production of these protective amino acids. Acclimated fish showed increased abundances of *Paracoccus zeaxanthinifaciens*, a bacterial species that produces the carotenoid zeaxanthin [59]. This pigment is a precursor for vitamin A synthesis and is vital for the health and growth of fishes [60]. Organisms within this genus are common in marine fish species [31, 61, 62] and may represent a natural probiotic. Mannose and fructose metabolism and biosynthesis of ansamycins increased in snook sampled from the bayous. Mannose competes against and prevents attachment of pathogens to the fish epithelium [63], and fructose increases susceptibility of microbes to antibiotics [64]. Ansamycins are antimicrobial compounds that are effective against a variety of fish pathogens, including *Mycobacterium*, *Francisella*, and *Aeromonas* [65], and improve fish survival during infections [66], suggesting that this pathway is activated against environmental microbes in natural ecosystems.

Bacteria play an essential role in nutrient cycling and xenobiotic degradation in aquatic ecosystems, and analysis of microbiota structure and gene expression is useful for environmental monitoring of water quality in natural and aquaculture systems [67, 68]. Patterns in microbiota structure and predicted function reveal that fish associate with bacterial taxa indicative of water quality. Microbiota of acclimated and wild juvenile snook contained higher abundances of four sulfur-metabolizing taxa (*Desulfococcus*, *Desulfosarcina* [69], *Vesicomyoscius okutanii* [70], and *Sulfurovum lithotrophicum* [71]). These bacterial groups are anaerobic, suggesting that the mucosal layer of fish harbors niches free from oxygen that allow for colonization by these microbes. Three genera positively correlated with SOD activity also relate to water quality. *Xenophilus* contains species that are resistant to toxic chemicals [72, 73], exposure to which results in oxidative stress in fish [53, 54]. High organic nitrogen levels enrich for *Armatimonas* (of the phylum Armatimonadetes, formerly candidate phylum OP-10) and *Bradyrhizobium* [74]. Although not specifically measured in this study, the nearest county water quality monitoring stations [75] (Station IDs WH-1 and HUD-3) indicated high total nitrogen levels during our sampling period as compared to historical averages (Online Resource 1). Water quality measurements from other agencies indicate organic enrichment in these bayous [76] (Waterbody IDs FL1936 and FL1953), and our results reflect this data. Acclimated fish harbored higher abundances of *Acinetobacter venetianus*, a known alkane-degrader [77], and predicted function of wild and acclimated microbiota showed patterns similar to those identified by Brown-Peterson et al. [58] within the gill microbiota of southern flounder *Paralichthys lethostigma* exposed to weathered crude oil-contaminated sediments. These results suggest the potential for fish-associated bacterial taxa to act as biomarkers for water quality in aquatic ecosystems.

## Conclusions

This study provides the first investigation into the adaptation of fish external microbiota to rapid movements between ecosystems such as those experienced in stock enhancement efforts. Results indicate that, within 2 days in predator-free enclosures, bacterial richness and evenness increased from initial captive measurements and microbiota structure changed significantly to reflect communities associated with wild fish, including differences identified between geographic locations. While captive fish harbored higher abundances of potential opportunistic pathogens, adaptation to the wild excluded these microbes from the external microbiota. Bacterial communities associated with fishes provide insight into water quality within the ecosystem, including the presence of organic enrichment and chemical contaminants. Overall, the skin-associated microbiota was able to quickly adapt to the wild environments, suggesting that bacteria, which play a vital role in health and disease resistance in their hosts, aid in acclimation of fishes to a new environment.

## Electronic Supplementary Material

Online Resource 1 Historical total nitrogen levels measured in left, Hudson bayou, and right, Whitaker bayou as obtained from www.sarasota.wateratlas.usf.edu. No data was available for Whitaker bayou from June 2008 through April 2012. Colored dots indicate sampling periods in this study (yellow, July 2016; blue, August 2016; green, September 2016; red, October 2016) (PDF 60 kb)
