# Supplementary figures and images for: Shifts in the Skin-Associated Microbiota of Hatchery-Reared Common Snook Centropomus undecimalis During Acclimation to the Wild
Source: Microb Ecol. 2018 Sep 6;77(3):770–81. doi: 10.1007/s00248-018-1252-7 (PMC6469608; doi:10.1007/s00248-018-1252-7)

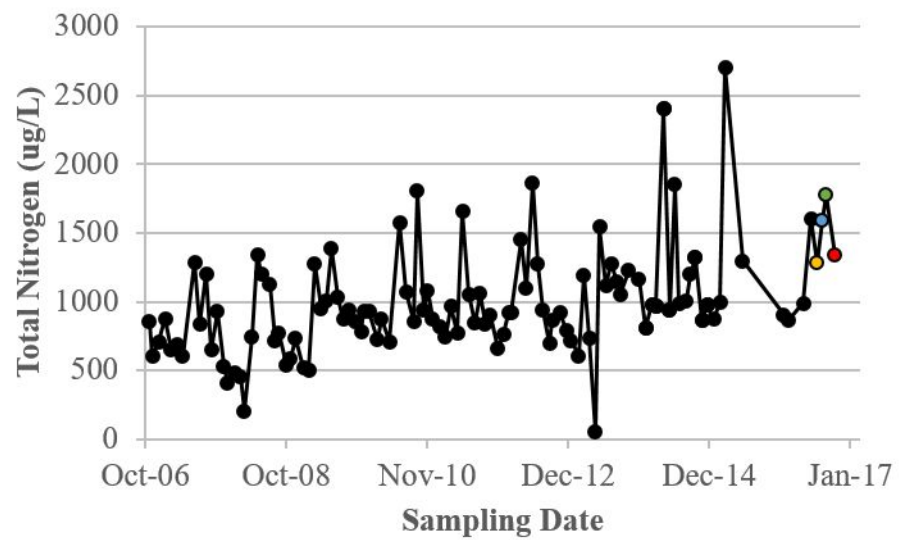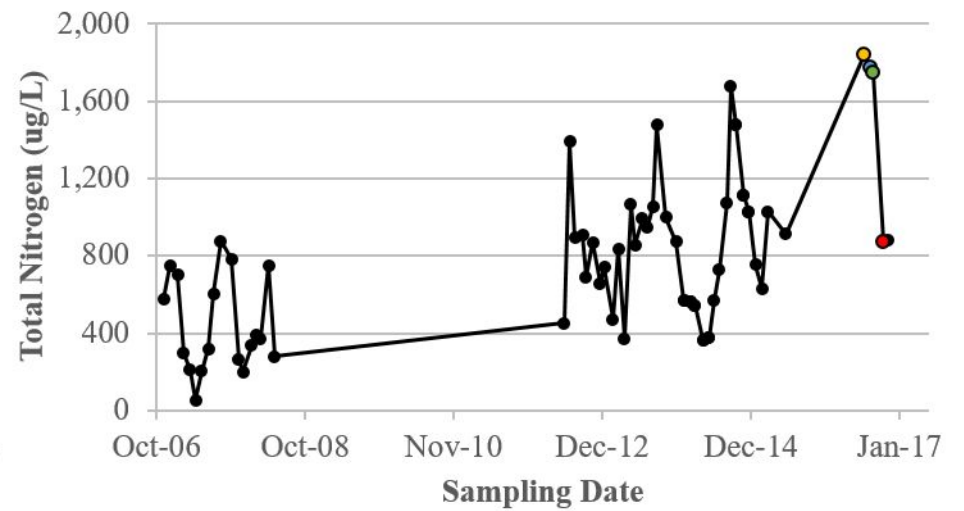

Online Resource 1

Supplement: Supplementary file 1 — Historical total nitrogen levels measured in left, Hudson bayou, and right, Whitaker bayou as obtained from www.sarasota.wateratlas.usf.edu. No data was available for Whitaker bayou from June 2008 through April 2012. Colored dots indicate sampling periods in this study (yellow, July 2016; blue, August 2016; green, September 2016; red, October 2016) (PDF 60 kb) [file 248_2018_1252_MOESM1_ESM.pdf]
